# A resource of longitudinal RNA-seq data of Holstein cow rumen, duodenum, and colon epithelial cells during the lactation cycle

**DOI:** 10.1186/s12863-025-01295-5

**Published:** 2025-01-27

**Authors:** Yahui Gao, George E. Liu, Li Ma, Cong-jun Li, Ransom L Baldwin

**Affiliations:** 1 Animal Genomics and Improvement Laboratory, BARC, Agricultural Research Service, USDA, Beltsville, MD 20705 USA; 2 Department of Animal and Avian Sciences, University of Maryland, College Park, MD 20742 USA; 3 State Key Laboratory of Livestock and Poultry Breeding, Guangdong Provincial Key Lab of Agro-Animal Genomics and Molecular Breeding, College of Animal Science, South China Agricultural University, Guangzhou, 510642 China

**Keywords:** Holstein cattle, Gene expression, Gastrointestinal tract tissues, Lactation, Functional genome, Molecular genetics

## Abstract

**Objective:**

As one of the most important ruminant breeds, Holstein cattle supply a significant portion of milk and dairy for human consumption, playing a crucial role in agribusiness. The goal of our study was to examine the molecular adaptation of gastrointestinal tissues that facilitate milk synthesis in dairy cattle.

**Data description:**

We performed RNA-seq analysis on epithelial cells from the rumen, duodenum, and colon at eight different time points: Days 3, 14, 28, 45, 120, 220, and 305 in milk, as well as the dry period. Samples were taken from five multiparous dairy cows as biological replicates per tissue per stage, except for Days 14 and 28, for which the sample size was three. These tissues each serve critical and distinct roles in the digestion and absorption of nutrients and are all vital for providing the necessary substrates required for milk production. Understanding the intricate connections between the tissues involved in providing nutrients necessary to support milk synthesis and their role in digestion can deepen the understanding of lactation physiology. This resource aims to deliver in-depth insights into cattle lactation, highlighting the distinct traits of gastrointestinal tissues and illuminating the intricate transcriptomic dynamics throughout the lactation period.

## Objective

The U.S. is the largest producer and exporter of milk protein globally. Holstein cattle are one of the major ruminants that supply milk and dairy products to the human diet and agribusiness [1]. The lactation cycle is the time interval between one calving and the next, which can be divided into 4 phases: the early (day D0-120), mid (D120-240), and late lactation (D240-305) (each spanning roughly 120 days) and the dry period (which could last as long as 65 days). Dairy cattle lactation is closely linked to varied nutrient needs essential for milk synthesis. Thus, milk production is a typical dynamic process that varies with time [2], during which the epithelial cells of the rumen and digestive tract must respond to metabolic reprogramming in a coordinated manner. The growth of the absorptive surface area is a well-documented phenomenon [3]. Still, the functional genomic changes in the epithelia of the rumen and other gastrointestinal tract tissues are less well-studied [4–6]. In particular, important information related to the dynamics of the transcriptomic activities over the full lactation period is lacking.

To address this question, epithelia from the rumen, duodenum, and colon were collected from Holstein cows. Samples were collected on D3, 14, 28, 45, 120, 220, and 305, which represented the four lactation phases: the early, mid, and late lactation and the dry period, respectively. RNA-seq and bioinformatics analyses were then performed to profile the changes in transcriptomes (Fig. 1 [7]) .This comprehensive dataset delivers stage- and tissue-specific transcriptome assessments of the cattle gastrointestinal tract tissues. This dataset could serve as a valuable resource for researchers aiming to enhance economically significant traits in cattle, including milk yield, feed efficiency, and overall health.

## Data description

### Animal collection and tissue preparation

The USDA ARS BARC research dairy herd is representative of the U.S. Holstein population and, as such, serves as a great model for this work. We gathered 108 samples of colon, duodenum, and rumen tissues from eight lactation stages (D3, D14, D28, D45, D120, D220, D305, and Dry), with each stage including three to five replicates (Table S1). Briefly, cows were surgically fitted with both a rumen fistula and a duodenal sampling cannula. Grab biopsies were used to collect rumen epithelial tissue (papillae) without requiring total rumen evacuation. Duodenal biopsies were performed with sterile biopsy forceps and a Pentax EC-383IL camera, inserted via the duodenal cannula, while colonic tissue was obtained using the same tools, inserted through the anus. Following the isolation of the three gastrointestinal tissues- colon, duodenum, and rumen- the samples underwent a series of saline rinses. Following overnight incubation at 4 °C in RNAlater^®^ Solution to facilitate thorough penetration, the samples were stored at −80 °C.

### RNA-seq library construction and sequencing

RNA extraction was performed using TRIzol (#15596026, Thermo Fisher Scientific), with concentration quantified via a Qubit^®^ RNA Assay Kit on a Qubit^®^ 2.0 Fluorometer (Life Technologies, USA). The integrity of the RNA was analyzed using a Bioanalyzer 2100 system (Agilent Technologies, USA). Rumen tissue samples underwent RNA isolation, quality control, library preparation, and sequencing at Admera Health LLC (South Plainfield, NJ). Using paired-end mode (2 × 150 bp reads), sequencing was performed on the Illumina HiSeq 2500 platform (Illumina, San Diego, CA, USA).

Sample information and RNA-seq read statistics can be found in the metadata presented in Table S1 and Fig. 2 [7]. FastQC (v0.12.1) was used to determine the quality of the raw RNA-seq data. Figure 2 presents a representative FastQC report, where Fig. 2A and b [7] demonstrate that the reads had consistently high-quality values. The GC content distribution mirrored the theoretical distribution, which confirms that the samples were uncontaminated (Fig. 2C [7]), . A peak at 150 bp in the sequence length distribution matched the expected fragment sizes of the RNA-seq libraries (Fig. 2D [7]), . Read quality was assessed using the geneBodyCoverage.py script from RseQC (v5.0.1), with no notable 5’ or 3’ end bias detected (Fig. 2E [7]), .

### Bioinformatics analyses

Trimmomatic (v0.39) [8] was used to remove adaptors and low-quality reads with parameters TruSeq3-PE.fa:2:30:10, LEADING:3, TRAILING:3, SLIDINGWINDOW:4:15, and MINLEN:36. The ARS-UCD1.2 [9] reference genome was indexed using HISAT2-build, and then the clean reads were aligned using HISAT2 (v2.2.1) [10]. Each sample had an average of 20.42 million input reads, ranging from 16.78 to 25.74 million, and an average unique alignment rate of 97.06%, with a range of 94.96–98.64% (Fig. 3 [7]).

## Limitations

While this study focuses on Holstein cattle, providing valuable insights into a major dairy breed, the findings may not fully extend to other breeds with different genetic backgrounds or environmental adaptations. Future studies could expand this research by including additional cattle breeds, offering a broader understanding of gene expression across diverse populations.

Our use of short-read RNA sequencing, combined with tools like Samtools (v1.12) [11], StringTie (v2.2.1) [12], and featureCounts (v2.0.3) [13], successfully captured key gene expression dynamics (Fig. 4A and C [7]). However, incorporating long-read sequencing technologies in future research could reveal more complex transcript structures and novel isoforms, further enriching the understanding of cattle transcriptomics. The tissue-specific variability highlighted by our principal component analysis (PCA) provides an important foundation for exploring gene regulation across different physiological stages PCA (Fig. 4B [7]). Expanding tissue diversity and sampling across more time points could uncover additional layers of gene regulation and inter-tissue interactions, further deepening our insights. Finally, while we identified differentially expressed genes (DEGs) using DESeq2 (v1.30.0) [14] under stringent thresholds (adjusted P-value ≤ 0.05, absolute log2 fold change ≥ 0.1 in Fig. 5 [7]), further functional validation through techniques like qPCR or proteomics would strengthen and confirm our findings Table 1.

Overall, this study offers a robust analysis of Holstein cattle gene expression during lactation and sets the stage for future research. Expanding on these findings will help further advance our understanding of cattle genetics, with potential applications for improving breeding strategies and dairy production efficiency.

**Table 1:** Overview of data files/data sets

Label	Name of data file/data set	File types (file extension)	Data repository and identifier (DOI or accession number)
Data file 1	Raw RNA-seq data	SRA file (.fastq)	NCBI SRA under the SRA ID SRP441033 [15]
Data file 2	Figure 1. The workflow for this study comprised data collection and technical analysis.	Document file (.pdf)	Figshare, 10.6084/m9.figshare.27275463.v1 [7]
Data file 3	Figure 2. RNA-seq quality check summary.	Document file (.pdf)	Figshare, 10.6084/m9.figshare.27275463.v1 [7]
Data file 4	Figure 3. The mapping rate and numbers of mapping reads with different lactation stages across three tissues.	Document file (.pdf)	Figshare, 10.6084/m9.figshare.27275463.v1 [7]
Data file 5	Figure 4. Summary based on gene expression.	Document file (.pdf)	Figshare, 10.6084/m9.figshare.27275463.v1 [7]
Data file 6	Figure 5. The number of differentially expressed genes between all possible pairwise comparison groups.	Document file (.pdf)	Figshare, 10.6084/m9.figshare.27275463.v1 [7]
Data file 7	Table S1. RNA-seq statistics.	Spreadsheet (.xlsx)	Figshare, 10.6084/m9.figshare.27275463.v1 [7]

## Data Availability

The RNA-Seq data were deposited in the NCBI Sequence Read Archive (SRA) under the accession number PRJNA979929.

## Abbreviations

PCA: Principal components analysis
DEGs: Differentially expressed genes
